# Nitrous oxide-induced polyneuropathy, pancytopenia and pulmonary embolism: a case report

**DOI:** 10.1186/s13256-023-04075-w

**Published:** 2023-08-17

**Authors:** Guillaume Parein, Benjamin Bollens

**Affiliations:** Physical and Rehabilitation Medicine Department, CHU Ambroise Paré, Mons, Wallonie, Belgium

**Keywords:** Case report, Nitrous oxide abuse, Polyneuropathy, Pulmonary embolism, Pancytopenia

## Abstract

**Background:**

Nitrous oxide is a medical and household gas that has seen its use drift to recreational purpose among the young population in recent years. Significant neurological, hematological and psychiatric side effects, generally related to an induced functional vitamin B12 deficiency, have been described separately in the literature.

**Case report:**

A 22-year-old woman of North African origin experienced an exceptional combination of polyneuropathy, bilateral pulmonary embolism and severe pancytopenia related to vitamin B12 deficiency and hyperhomocysteinemia induced by recreational nitrous oxide use. After treatment with vitamin B12 supplementation and intensive rehabilitative management, the patient progressively regained the ability to walk and her biological parameters gradually returned to normal. The pathophysiological mechanisms related to a decrease in vitamin B12 activity are the reduction of products needed for synthesis of deoxyribonucleic acid, carbohydrate or fatty acids, and the increase of hyperhomocysteinemia. Other mechanisms involving a direct action of N2O are also suspected.

**Conclusion:**

This case report brings elements to support our knowledge about pathological pathway, recovery and prognosis of recreational N2O abuse complications. The general and medical population should be aware to the serious consequences of this type of consumption.

## Introduction

Nitrogen peroxide (N2O) is a colourless and odourless inhaled substance that has been classically used in anaesthesia for over 150 years for its analgesic and anxiolytic properties [1]. Unfortunately, its use has been derived for recreational purposes in recent decades among the young population wishing to exploit its euphoric effect. Young people ingest N2O from bottles intended for domestic use, which are easily available on the market [2]. Many side effects related to chronic intake have been reported with some dramatic consequences in this population. Among the reported side effects, we note psychiatric disorders, damage to the central and peripheral nervous systems, hematological disorders, thromboembolic phenomena, and others [2, 3]. These side effects generally result from an induced vitamin B12 deficiency.

Here we describe the case of a young patient who exceptionally presented a simultaneous combination of several side effects, namely polyneuropathy, pulmonary embolism, and pancytopenia.

## Presentation of the case

A 22-year-old woman of North African origin was brought to the emergency department for confusional syndrome and ataxia after being found at home in this condition and surrounded by nitrous oxide cylinders. She had no relevant medical history or familial history. She did not take any medication or contraceptives. The initial heteroanamnesis reported a depressive state that had been evolving for a few weeks. The patient's main complaints were headache, dyspnoea and pain in both knees following a fall. She had no chest pain, no cough, no expectoration, no abdominal pain, and no urinary complaints.

On clinical examination, the patient presented with an increased heart rate of 100 beats/min, and pyrexia at 38.4 °C. The other parameters were normal. Her hygiene was poor, and she was clinically dehydrated. She was eupnoeic at rest. Cardiopulmonary auscultation was unremarkable. No signs of acute abdomen or visceral mass were noted. There were no impairments in the lower limbs. Neurologically, she had L1-level paraparesis with hypoesthesia interesting the epicritic modality. Protopathic sensitivity, nociception and proprioception were preserved. The deep tendon reflexes were average and symmetrical. There were no pyramidal or extra-pyramidal signs. There were no meningeal signs. The cerebellar tests were correctly performed. The examination of the cranial nerves was normal. She had no dysarthria or phasic disorder. No bladder or sphincters disorders were found.

Her initial biology showed an inflammatory syndrome with non-megaloblastic pancytopenia (Table 1). Vitamin B12 was under 148 ng/l. Search for alcohol or benzodiazepine was negative. Urine analysis was negative for any illicit drug but revealed leukocyturia with bacteriuria. The urine culture was later positive for E. coli. The biological assessment was completed by the search for pro-thrombotic factors, which showed hyperhomocysteinemia (207.1 µmol/l [nl < 10]). Methylmalonic acid was also elevated to 919.00 µg/l. Biermer's disease was ruled out based on a normal gastroscopy performed a few months earlier and the absence of intrinsic factor and parietal cell antibodies in the blood. Finally, a myelogram was performed and excluded any sign of acute leukemia or macrophage activation syndrome (MAS). The marrow was of normal richness, without excess blasts whose cell morphology was compatible with a vitamin B12 deficiency.

**Table 1:** Evolution of biological data

Time after presentation (days)	1	2	7	18	74
Hemoglobin [g/dl] (nl: 11.5–15)	7.5	7.7	7 .6	8.9	12.4
Hematocrit [%] (nl: 34.4–44.6)	21.7	21.8	22.6	27.4	37.8
MCV [fl] (nl: 75–96)	83.5	82.6	86.6	94.5	92.2
Leucocytes [10^3/µL] (nl: 3.8–11.4)	2.12	2.01	6.27	4.81	5.81
Neutrophils [10^3/µL] (nl: 1.4–7.7)	0.17	0.28		2.14	2.71
Thrombocytes [10^3/µL] (nl: 150–445)	133	88	83	425	326
Reticulocytes [10^9/l] (nl: 20–100)	10.2	–	–	210.8	–
Haptoglobin [g/l] (nl: 0.35–2.5)	2.69	–	–	3.19	–
Schizocytes	Absents	–	–		–
Iron [µg/dl] (nl: 50–170)	18	–	–	40	38
Ferritin [µg/l] (nl: 4.63–204)	1240.14	–	–	256.98	31.69
Transferrin [g/l] (nl: 1.8–3.82)	1.54	–	–	2.34	2.67
Transferrin saturation [%] (nl: 15–50)	8	–	–	12	10
CRP [mg/l] (nl: 0–5)	316.6	297.1	63	12.1	–
Folic acid [µg/l] (nl: 3.1–20.5)	13,39	–	–	15.49	19.78
Vitamin B12 [ng/l] (nl: 187–883)	< 148	–	> 2000	> 2000	374
Homocysteine [µmol/L] (nl: < 10)	207.1	–	–	–	–
Methylmalonic acid [µg/l] (nl: < 47)	919	–	–	–	–

MCV: mean corpuscular volume

CRP: C-reactive protein

Nl: normal

A cranial CT-scan did not reveal any acute phenomenon or suspicious parenchymal or meningeal signs. A thoraco-abdominal CT-scan revealed a right lower lobar and a posterobasal segment of the left lower lobe pulmonary embolism, with no lesions at the abdominal level. The iconographic work-up was then completed by an MRI of the brain and spine, which confirmed the absence of brain or spinal cord injury. Electroneuromyography (ENMG) performed 2 weeks after the onset of the symptoms showed slowing of the motor conductions of the lower limbs with lengthening of the distal motor latencies, amplitude decease of the compound muscle action potential and conduction blocks. No abnormality of the sensory conductions was found in the lower limbs and the EMG of the upper limbs was normal (Table 2). To exclude a partial form of Guillain-Barré syndrome, a lumbar puncture was carried out to search an albumin-cytological dissociation, which was negative.

**Table 2:** Electroneuromyography parameters

2a Motor conduction study				
	Latency (ms)	Amplitude (mV)	MNCV (m/s)	F wave latency (ms)
Tibial nerve (right)				
Ankle—AHB	4.89	2.1	–	NR
Popliteal fossa—Ankle	18.0	0.3	32.0	–
Tibial nerve (left)				
Ankle—AHB	5.12	0.9	–	NR
Popliteal fossa—Ankle	17.5	0.55	32.3	–
Peroneal nerve (right)				
Ankle—EDB	6.08	0.33	–	NR
Fibula head—Ankle	18.8	0.21	28.3	–
Median nerve (right)				
Wrist—APB	3.58	9.7	–	26.3
Elbow—Wrist	7.38	9.6	51.3	–
Ulnar nerve (right)				
Wrist—ADM	2.75	8.9	–	27.9
Elbow (below)—Wrist	6.69	7.0	54.6	–

2b Sensitive conduction study			
	Latency (ms)	Amplitude (µV)	SNCV (m/s)
Sural nerve (right)	2.88	8.4	48.6
Median nerve (right)	3.04	25.9	59.2

ADM: abductor digiti minimi; AHB: Abductor hallucis brevis; APB: abductor pollicis brevis; EDB: Extensor digitorum brevis; MNCV: Motor nerve conduction velocity; NR: No response; SNCV: Sensitive nerve conduction velocity

In total, the patient presented with pancytopenia with febrile neutropenia, urinary sepsis, bilateral pulmonary embolism and paraparesis due to polyneuropathy.

The urinary sepsis was treated with empirical antibiotic therapy with piperacillin-tazobactam before a relay with amoxicillin-clavulanic acid 1 g 4 times a day for 1 week with a good evolution of the inflammatory syndrome. The pulmonary embolism was treated by therapeutic anticoagulation with low molecular-weight heparin (enoxaparin) 1 mg/kg twice a day before switching to dabigatran 150 mg twice a day for 6 months. A ventilation/perfusion pulmonary scintigraphy performed 3 months after the start of the treatment showed disappearance of the pulmonary embolism. Pancytopenia was treated by transfusion of 2 erythrocyte pellets with oral folic acid supplementation 4 mg once a day and intravenous vitamin B12 1 mg once a day for 10 days before intramuscular relay. Progressive correction of the three hematopoietic lineages was possible thanks to these treatments (Table 1).

Despite vitamin B12 supplementation, the patient still had significant paraparesis with gait disturbances and loss of autonomy. Four weeks after acute treatment, she was transferred to the neurological rehabilitation department. She received daily physiotherapy and occupational therapy for 2 h a day. The patient's motor testing progressively improved, except for persistent paresis of the levator muscles of the feet (Table 3a). This paresis was compensated by the placement of Liberty® foot lifts orthoses. After 6 weeks of rehabilitation, the patient recovered autonomous walking without technical or human assistance. The Functional Ambulatory Category (FAC) improved from 0 (non-functional ambulator) at the time of her admission in rehabilitation and to 5 (ambulator, independent) after rehabilitation (Table 3b). She also regained complete autonomy for activities of daily living. She finally left the hospital after 14 weeks of hospitalisation, including 10 weeks in the rehabilitation department.

**Table 3:** Functional evolution

3a Lower limbs muscle testing (MRC scale)							
Time after presentation (weeks)	1	4	6	8	10	12	33
Psoas							
Right	3	3	3	4	5	5	5
Left	3	3	3	4	5	5	5
Quadriceps							
Right	2	2	2	4	5	5	5
Left	1	1	1	3	5	5	5
Tibialis anterior							
Right	1	1	2	2	2	2	2
Left	0	0	1	1	1	1	2
Tibialis posterior							
Right	1	1			3	3	3
Left	0	0			2	2	5
Peroneus longus							
Right	1	1			1	1	5
Left	0	0			1	1	5
Triceps surae							
Right	4	4	4	4	4	4	5
Left	1	1	1	2	2	4	5
Extensor hallucis longus							
Right	2	2	2	2			
Left	0	0	1	1			

3b Evolution of walking ability (FAC)							
Time after presentation (weeks)	1	4	6	8	10	12	33
FAC	0	0	1	3	4	5	5

MRC scale: Medical Research Council scale

FAC: Functional Ambulation Categories

A follow-up ENMG performed 17 weeks after the onset of the neurological symptoms highlighted the absence of motor response of the tibial and fibular nerves bilaterally, the absence of the H reflex using the S1 roots, and the persistence of denervation signs at rest and decreased motor recruitment during voluntary contraction in the medial gastrocnemius and tibial anterior muscles. There was no abnormality in the upper limbs. We can therefore conclude to the persistence of severe signs of motor polyneuropathy in the lower limbs 4 months after the treatment onset.

A follow-up visit at 33 weeks showed that the patient still had weakness of the tibialis anterior muscle but had improved testing of the peroneus longus (Table 3a). In addition, she was no longer using her Liberty® foot lifts orthosis despite the persistence of a slight bilateral foot drop for social reasons. She had finally recovered a normal lifestyle and professional activity.

## Discussion

Pancytopenia, pulmonary embolism and polyneuropathy can be each caused by numerous separate conditions. In the present case report, it was first important to exclude the most probable origins for each of these conditions, which explains the extensive assessments that were carried out. In the absence of a diagnosis that better explains the onset of these conditions, the diagnosis of nitrous oxide-induced polyneuropathy, pancytopenia and pulmonary embolism was retained.

The occurrence of these conditions and their physiological pathway have already been described in the literature in the recent years. The novelty of our case is that this young patient experienced simultaneously severe pancytopenia, bilateral pulmonary embolism and polyneuropathy related to vitamin B12 deficiency and hyperhomocysteinemia induced by recreational nitrous oxide use. She completely recovered from pulmonary embolism and pancytopenia thanks to the adequate treatments. From a neurological point of view, she kept mild motor sequelae consisting in distal paraparesis after 8 months of evolution. She underwent rehabilitation and resumed an active lifestyle. The patient admitted consuming small N2O bottles or larger bottles of 2 kg 3 to 4 times a week before the incident.

### N2O toxicity: vitamin B12 deficiency

**Fig. 1:**
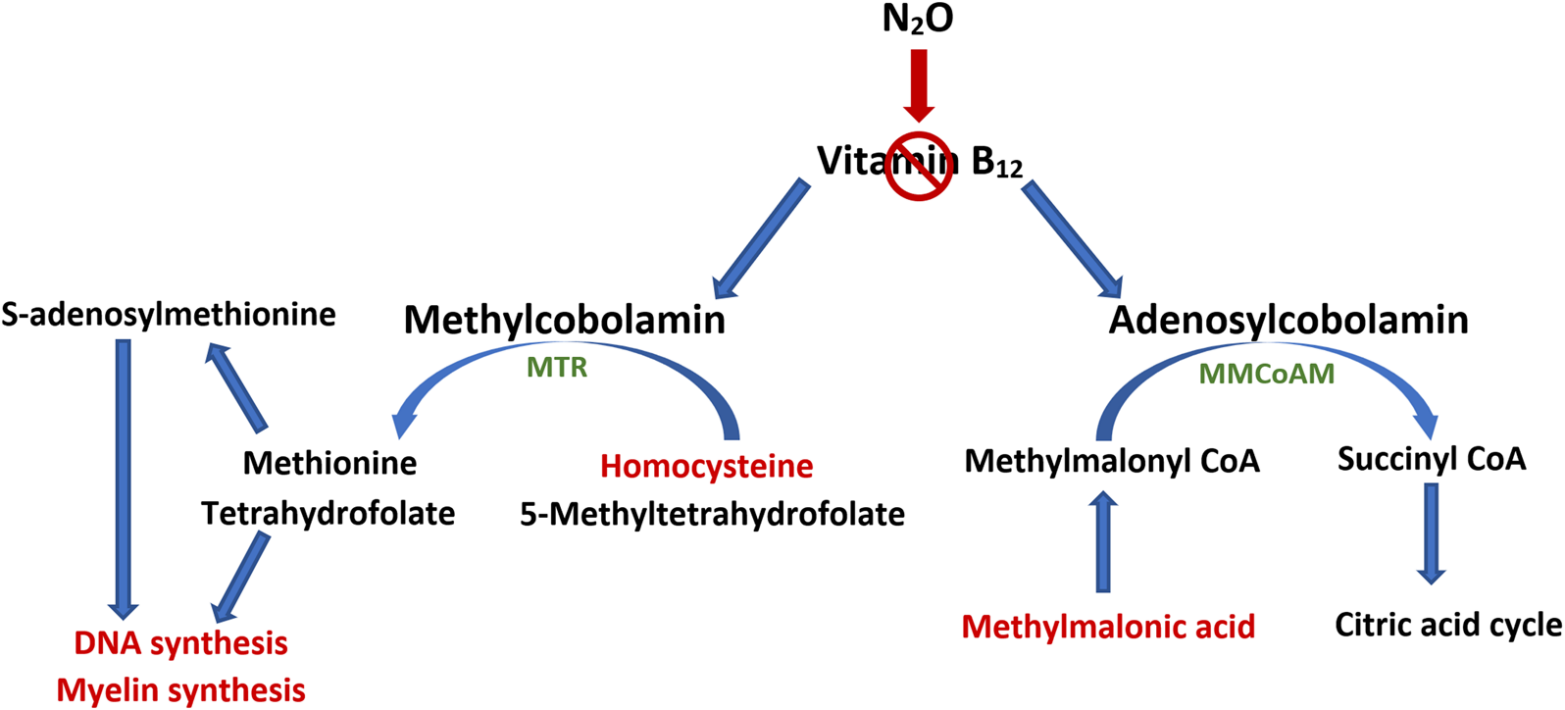
Diagram of the metabolism of vitamin B12 and its two active forms. Methylcobolamine is a cofactor of methionine synthetase (MTR) and adenosylcobolamine is a cofactor of methylmalonyl coenzyme A mutase (MMCoAM). MTR is responsible for the conversion of homocysteine and 5-methyltetrahydrofolate to methionine and tetrahydrofolate. MMCoAM is responsible for the conversion of methylmalonyl CoA to Succinyl CoA

Vitamin B12, also known as cobolamin, is present in the body in two active forms, methylcobolamin and adenosylcobolamin. These molecules act as cofactors for methionine synthetase (MTR) and methylmalonyl coenzyme A mutase (MMCoAM) respectively (Fig. 1). N2O is known to irreversibly oxidizes the cobalt ion of vitamin B12 from its 1+ active form to its 3+ and 2+ valences of inactive form, thereby leading to a functional B12 deficiency [4, 5]. A decrease in vitamin B12 is responsible for a decrease in the activity of these enzymes and consequently a decrease in their product associated with an increase in their substrate. While MTR activity was found to be rapidly inhibited by N2O exposure, MMCoAM activity is not affected by a short exposure effect but could decrease after a prolonged exposure [4]. A decrease in MTR activity results in an increase in homocysteine and a reduction in methionine and S-adenosylmethionine required for the methylation of phospholipids in myelin or the membrane of haematopoietic cells. Blockage of MTR also results in an inability to convert 5-methyltetrahydrofolate to tetrahydrofolate, thus impacting DNA synthesis. This blockage affects cells which undergo rapid turnover, such as blood cells. A decrease in MMCoAM activity results in an increase in methylmalonic acid and a decrease in Succinyl coenzyme A which is important in the synthesis of carbohydrates and fatty acid [4, 6]. This process explains the high levels of homocysteine and methylmalonic acid found in the patient. It should be noted that vitamin B12 can be normal, which does not exclude vitamin B12 deficiency. In this case, it is therefore necessary to rely on homocysteine and methylmalonic acid levels to confirm the diagnosis [7].

### Peripheral neuropathy

Peripheral neuropathies related to recreational use of N2O have already been described several times in the literature [8, 9]. Neurological symptoms are extremely frequent, and Oussalah et al. report the presence of at least one neurological symptom in 96% of patients who have used N2O recreationally [3]. The probability of developing neurological symptoms is directly proportional to the quantity of N2O consumed and inversely proportional to the age at the time of consumption [10]. As seen above, N2O-induced vitamin B12 deficiency leads to a decrease in the methionine and S-adenosylmethionine necessary for the methylation of myelin phospholipids. The myelination of neuronal axons is then ineffective, which explains our patient's peripheral neuropathy. Some authors emphasize the fact that there may also be a direct toxic component of N2O independently of the vitamin B12 metabolic pathway [11]. A cessation of N2O consumption, vitamin B12 supplementation and specific rehabilitative management are necessary to restore nerve conduction capacity leading to a recovery of walking. Orthoses may be used to combat vicious joint positions or to compensate for residual deficiencies (such as equinus of the foot and stepping in case of levator paresis).

#### Neurophysiologic features

Recent studies have analyzed the neurophysiological characteristics of polyneuropathies induced by voluntary N2O intake. They show that N2O abuse can cause motor and sensory neuropathies with several cases presenting only a motor neuropathy without conduction block [11–14]. Typical clinical features of the acute N2O neuropathy appear to be mixed axonal-demyelinating, affecting the lower limbs more than the upper limbs with length dependence of peripheral nerve injury [11, 13, 14]. The peroneal and tibial nerves seem to be the most frequently impacted nerves [13]. Concerning motor damage, one hypothesis is that motor neurons would suffer from damage related to oxidative stress induced by homocysteine, leading to excitotoxicity, increased DNA damage or ATP depletion resulting in neuronal death [12]. Higher homocysteine levels could also be associated with severe CMAP and SNAP impairment, indicating more severe axonal injuries [13].

In terms of prognosis, it seems that some patients recover completely while others retain motor impairments with generally difficulties mainly related to bilateral foot drop. However, the follow-up of patients in these studies remains quite heterogeneous, varying from 1 to 14 months [12, 14].

### Thrombotic events

Thrombotic events related to recreational NO2 use, although less frequent, have also been described in the literature. Pratt DN et al. were the first to report a case linking recreational N2O use with the development of venous thrombosis [15]. Oulkadi et al. conducted a systematic review in 2022 describing 14 cases of patients who developed thrombotic complications after recreational NO2 use. Of these, 4 involved the development of pulmonary embolism [16]. Again, this would be a consequence of the inactivation of vitamin B12 by N2O leading to an increase in homocysteine. Hyperhomocysteinemia is known to increase the risk of venous thrombosis compared to the population with normal homocysteinemia [17–19]. However, the mechanism of action remains unclear and this association tends to be controversial [20]. However, we argue that in our patient's case, high homocysteine levels have contributed to pulmonary embolism as no other major risk factor was found. Treatment of these complications usually consists in anticoagulant therapy with either therapeutic dose heparin or direct oral anticoagulant until resolution of the thrombosis. In addition, vitamin B12 supplementation appears to be required to restore normal homocysteine levels.

### Hematological effects

Oussalah et al. demonstrated in their 2019 systematic review that, in addition to neurological damage, patients who had suffered N2O poisoning had at least one haematological alteration in 71.7% of cases, the 3 main alterations are a drop in haemoglobin level (55.8%), a drop in haematocrit (52.4%) and macrocytosis (41.8%) [3]. Vitamin B12 deficiency is well recognised as an aetiology of pancytopenia with several cases reported in the literature describing an acute leukaemia-like clinic [21–23]. To date, only two cases of pancytopenia due to vitamin B12 deficiency induced by recreational use of N2O have been reported [24, 25]. It should be noted that in most cases macrocytosis is associated with anaemia. In the present case report, the anaemia is particularly significant with normal mean corpuscular volume. We hypothesize that the concomitant iron deficiency caused by the inflammatory syndrome related to the urinary sepsis amplified the anaemia and reduced the mean corpuscular volume. The treatment of pancytopenia in this context includes one or more transfusions of figurative elements as well as a correction of vitamin B12 deficiency by supplementation. In this case, after the correction of vitamin B12 level, the three haematopoietic lineages were quickly readjusted, alongside an increase in reticulocytes, showing the resumption of haematopoietic synthesis.

## Conclusion

This case report explores the toxic effects of chronic N2O intoxication. To our knowledge, this is the first case to report simultaneous occurrence of peripheral neuropathy, bilateral pulmonary embolism and pancytopenia. Cessation of N2O exposure and vitamin B12 supplementation are essential for the treatment of each of these conditions in addition to their specific treatments. This case also describes the evolution of peripheral neuropathy, contributing to our knowledge of the prognosis for functional recovery in this type of injury.

Our report presents a few limitations. Firstly, several caregivers attended the patient at different points in her medical history, which may contribute to a loss of information or bias due to inter-observer variability in the clinical examinations performed. Secondly, the patient's history remains unclear, and it's possible that some anamnesis information has been omitted by the patient, whether voluntarily or not, which may affect the interpretation of the results.

This case allows medical practitioners to be aware of the need to look for N2O consumption when faced with this type of clinical presentation.

## Data Availability

The data for this case report are located at CHU Ambroise Paré, Mons, Belgium and are available from the corresponding author on reasonable request.

## Abbreviations

N2O: Nitrous oxide
CRP: C-reactive protein
MCV: Mean corpuscular volume
ENMG: Electroneuromyography
MRC: Medical Research Council
FAC: Functional ambulation categories
MTR: Methionine synthetase
MMCoAM: Methylmalonyl coenzyme A mutase
DNA: Deoxyribonucleic acid
ATP: Adenosine triphosphate
CMAP: Compound muscle action potential
SNAP: Sensory nerve action potential
